# Myocardial infarction with non-obstructive coronary arteries: a focus on vasospastic angina

**DOI:** 10.1007/s12471-019-1232-7

**Published:** 2019-01-28

**Authors:** M. A. Beijk, W. V. Vlastra, R. Delewi, T. P. van de Hoef, S. M. Boekholdt, K. D. Sjauw, J. J. Piek

**Affiliations:** 1 Academic Medical Centre, University of Amsterdam, Amsterdam, The Netherlands; 2 Medisch Centrum Leeuwarden, Leeuwarden, The Netherlands

**Keywords:** Vasospastic angina, Myocardial infarction, Coronary artery disease, Non-obstructive coronary atherosclerosis

## Abstract

Vasospastic angina (VSA) is considered a broad diagnostic category including documented spontaneous episodes of angina pectoris produced by coronary epicardial vasospasm as well as those induced during provocative coronary vasospasm testing and coronary microvascular dysfunction due to microvascular spasm. The hallmark feature of VSA is rest angina, which promptly responds to short-acting nitrates; however, VSA can present with a great variety of symptoms, ranging from stable angina to acute coronary syndrome and even ventricular arrhythmia. VSA is more prevalent in females, who can present with symptoms different from those among male patients. This may lead to an underestimation of cardiac causes of chest-related symptoms in female patients, in particular if the coronary angiogram (CAG) is normal. Evaluation for the diagnosis of VSA includes standard 12-lead ECG during the attack, Holter monitoring, exercise testing, and echocardiography. Patients suspected of having VSA with a normal CAG without a clear myocardial or non-cardiac cause are candidates for provocative coronary vasospasm testing. The gold standard method for provocative coronary vasospasm testing involves the administration of a provocative drug during CAG while monitoring patient symptoms, ECG and documentation of the coronary artery. Treatment of VSA consists of lifestyle adaptations and pharmacotherapy with calcium channel blockers and nitrates.

## Introduction

The vast majority of acute myocardial infarction (AMI) patients have obstructive coronary artery disease (CAD) (i. e. ≥50% stenosis) at coronary angiography (CAG) and well-established therapeutic guidelines are available, often involving coronary revascularisation. However, 1–14% of AMI occur in the absence of obstructive CAD [1, 2]. Non-obstructive CAD in patients presenting with symptoms and ST-segment deviation suggestive of ischaemia does not preclude an atherothrombotic aetiology, as thrombosis can be a dynamic phenomenon with a non-obstructive atherosclerotic plaque. The diagnosis of myocardial infarction with non-obstructive coronary atherosclerosis (MINOCA) should be considered a ‘working diagnosis’ and its underlying cause should be investigated (Tab. 1 and 2).

**Table 1:** Diagnostic criteria for myocardial infarction with non-obstructive coronary artherosclerosis and vasospastic angina

*MINOCA diagnostic criteria elements*	
1	AMI criteria, including:
	(a) Positive cardiac biomarker: defined as a rise and/or fall in serial levels, with at least one value above the 99th percentile upper reference limit and
	(b) Corroborative clinical evidence of infarction, including any of the following:
	– i. Ischaemic symptoms (chest pain and/or dyspnoea)
	– ii. Ischaemic ECG changes (new ST-segment changes or LBBB)
	– iii. New pathological Q waves
	– iv. New loss of viable myocardium on myocardial perfusion imaging or new RWMA
	– v. Intracoronary thrombus evident on angiography or at autopsy
2	Absence of obstructive CAD on angiography (defined as no lesions ≥50%)
3	No clinically apparent cause for the acute presentation
*Vasospastic angina diagnostic criteria elements*	
1	Nitrate-responsive angina—during spontaneous episode, with at least one of the following:
	(a) Rest angina—especially between night and early morning
	(b) Marked diurnal variation in exercise tolerance—reduced in morning
	(c) Hyperventilation can precipitate an episode
	(d) Calcium channel blockers (but not beta-blockers) suppress episodes
2	Transient ischaemic ECG changes—during spontaneous episode, including any of the following in at least two contiguous leads:
	(a) ST-segment elevation ≥0.1 mV
	(b) ST-segment depression ≥0.1 mV
	(c) New negative U waves
3	Coronary artery spasm—defined as transient total or subtotal coronary artery occlusion (>90% constriction) with angina and ischaemic ECG changes either spontaneously or in response to a provocative stimulus (typically acetylcholine, ergonovine or hyperventilation)

*AMI* acute myocardial infarction, *CAD* coronary artery disease, *ECG* electrocardiogram, *LBBB* left bundle branch block, *RWMA* regional wall motion abnormality

**Table 2:** Mechanisms of myocardial infarction with non-obstructive coronary atherosclerosis

*Clinical disorder*		
1	Epicardiac coronary disorders (MI type 1)	(a) Atherosclerotic plaque rupture
		(b) Ulceration
		(c) Fissuring
		(d) Erosion or coronary dissection with non-obstructive CAD
2	Imbalance between oxygen supply and demand (MI type 2)	(a) Coronary embolism
		(b) Coronary artery vasospasm
3	Coronary endothelial dysfunction (MI type 2)	(a) Coronary microvascular dysfunction
4	Myocardial causes	(a) Cardiomyopathy
		– i. Takotsubo syndrome
		– ii. Dilated
		– iii. Hypertrophic
		(b) (Peri)-myocarditis
		(c) Myocardial trauma or injury
		(d) Tachyarrhythmia-induced infarct
5	Non-cardiac causes	(a) Renal impairment
		(b) Pulmonary embolism

*CAD* coronary artery disease, *MI* myocardial infarction

**Fig. 1:**
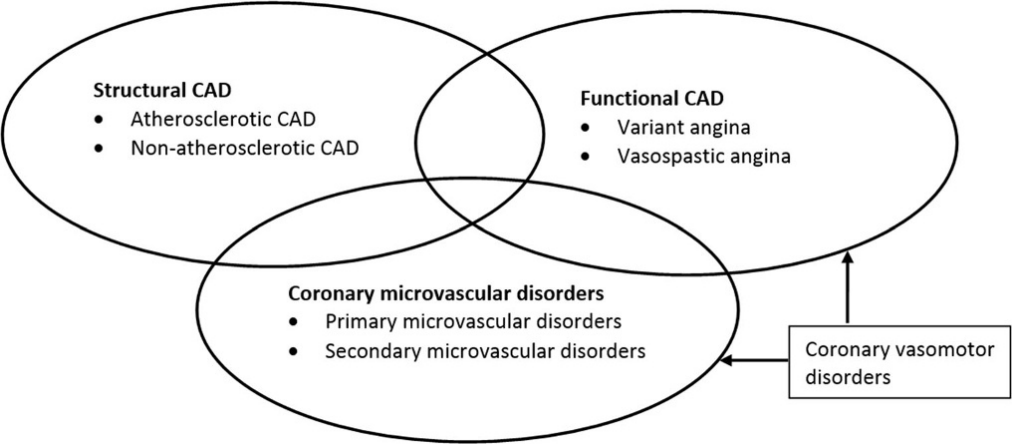
Ischaemic heart disease (*CAD* Coronary artery disease)

Vasospastic angina (VSA), basically synonymous with the terms Prinzmetal’s angina and variant angina, is an important functional cardiac disorder leading to type 2 myocardial infarction [3]. The term VSA is considered a broad diagnostic category including documented spontaneous episodes of angina pectoris produced by coronary epicardial vasospasm (EV) and/or coronary microvascular dysfunction (CMD) due to microvascular spasm as well as angina pectoris induced by provocative coronary vasospasm testing. The diagnostic criteria for VSA as proposed by the Coronary Vasomotion Disorders International Study Group (COVADIS) [4] are summarised in Tab. 1. Although VSA may co-exist with coronary microvascular disorders and/or structural CAD (Fig. 1), it is a clinical entity that involves hyperreactivity of the epicardial arteries to vasoconstrictor stimuli [5]. The importance of diagnosing VSA relates to: (1) the major adverse events associated with this disorder including AMI, syncope due to arrhythmia, and sudden cardiac death (SCD) [6–8], and (2) the potential to prevent adverse events by the use of calcium channel blockers and nitrates and avoiding potential vasospasm precipitants (e. g. vasoconstrictors). This article aims to provide an overview of the clinical characteristics, diagnostic tests, and treatment for VSA patients. PubMed and Embase were searched for relevant articles focusing on the following terms: ‘coronary artery vasospasm’, ‘vasospastic angina’, ‘Prinzmetal angina’, ‘non-obstructive’, and ‘myocardial infarction’. This article will focus on VSA, either EV or microvascular vasospasm, and will not fully elaborate on CMD in all its subforms.

## Clinical manifestations of vasospastic angina

The prevalence of VSA remains largely unknown but ranges between 3 and 95% of all MINOCA patients depending on the stimuli used to trigger vasospasm, definitions of vasospasm, and ethnic background [9]. The hallmark feature of VSA is rest angina, which promptly responds to short-acting nitrates; however, VSA can present with a great variety of symptoms, such as silent myocardial ischaemia, stable angina, acute coronary syndrome or SCD [10, 11]. Patients with VSA typically experience angina at rest, during the night or early in the morning, and this can be precipitated by hyperventilation [10, 12]. A study systematically performing invasive provocative vasospasm testing in 1,089 consecutive patients (excluding patients with spontaneous spasm, left main narrowing or severe three-vessel disease) showed that EV was present in 38% of patients with angina only at rest, 14% of those with angina at rest and during exercise, 4% with only exertional angina, 1% with atypical chest pain, 20% of patients with a recent AMI, 6% of patients with an ‘old’ myocardial infarction, and 0% of patients with congestive cardiomyopathy [13]. Importantly, VSA can be induced by exercise, especially in the morning [14]. Even though brief episodes of vasospasm can be asymptomatic, they may generate silent myocardial ischaemia [12]. Moreover, various arrhythmias are associated with VSA even in the absence of angina, including sinus bradycardia, sinus arrest with or without junctional escape beats, complete atrioventricular block, paroxysmal atrial fibrillation, ventricular tachycardia, ventricular fibrillation and asystole [11, 15, 16]. It is noteworthy that VSA-related SCD is most frequently related to bradyarrhythmia rather than tachyarrhythmia [17].

The prognosis of patients diagnosed with VSA is variable and depends on the degree of vasospasm. A novel scoring system, the Japanese Coronary Spasm Association (JCSA), may provide a comprehensive risk assessment and prognostic stratification for VSA patients [18]. Although not validated in Caucasian patients, the JCSA score includes predictors of major adverse cardiac events (MACE): history of out-of-hospital cardiac arrest (OHCA) (4 points); multivessel EV, smoking, angina at rest alone, coronary stenosis (2 points each); ST-segment elevation during angina, and beta-blocker use (1 point each). Patients can be categorised as low risk (score 0–2), intermediate risk (score 3–5) or high risk (score ≥6), resulting in an incidence of MACE of 2.5%, 7.0%, and 13%, respectively at a median follow-up of 32 months.

### Risk factors and pathogenesis

VSA is more prevalent among females than males [19, 20]. The importance of recognising sex differences is enhanced by the fact that VSA in female patients can present with different symptoms than those in male patients. This may lead to an underestimation of cardiac causes of chest-related symptoms in female patients, in particular if the CAG is normal. In the largest European study including 1,379 consecutive patients with stable angina and unobstructed coronary arteries, acetylcholine (ACH) tests were performed, 59% had a positive ACH test (33% for CMD, 26% for EV) [19]. A positive ACH test was more common in females (70% vs 43%; *p* < 0.001). In a multivariable logistic regression model the sex difference was statistically significant with a female–male odds ratio for CMD and EV of 4.2 (95% confidence interval: 3.1–5.5; *p* < 0.001) and 2.3 (95% confidence interval: 1.7–3.1; *p* < 0.001), respectively.

In general, most patients with VSA are diagnosed between 40 and 70 years of age. While smoking and high-sensitivity C‑reactive protein are risk factors for VSA [21, 22], it can be precipitated by many factors such as physical and/or emotional stress, alcohol consumption, magnesium deficiency, and the administration of stimulant drugs (cocaine, amphetamines, etc.), sympathomimetic agents (epinephrine, norepinephrine, etc.), parasympathomimetic agents (methacholine, pilocarpine, etc.), vasoconstrictor agents (beta-blockers, anti-migraine drugs, etc.) and ergot alkaloids (ergonovine (ER), ergotamine, etc.) [23–26]. Genetic mutations were found to be associated with VSA in genes coding for proteins for adrenergic or serotoninergic receptors, angiotensin-converting enzyme, and inflammatory cytokines [11]. Polymorphisms of paraoxonase I gene and mutations or polymorphisms of the endothelial NO synthase gene are also found, albeit NO gene polymorphisms are only found in one-third of VSA patients [11]. The pathophysiology of VSA is the result of the interaction of two components: (1) hyperreactivity of vascular smooth muscle cells (VSMCs; localised or diffuse) and (2) a transient vasoconstrictor stimulus acting on the hyperreactive VSMCs [9]. The main cause of VSMC hyperreactivity seems to be enhanced Rho kinase activity [1]. In summary, considering the abundance of triggers and the various vasoconstrictors that can be used to provoke coronary vasospasm, this suggests it is not the consequence of a single receptor pathway problem, but rather multifactorial.

## Evaluation of patients with vasospastic angina

### Non-invasive evaluation

Non-invasive, non-pharmacological evaluation for the diagnosis of VSA includes standard 12-lead ECG during an attack, Holter monitoring, and exercise testing [20]. ECG changes are related to the severity of vasospasm. EV is more frequently associated with ST-segment depression rather than ST-segment elevation, indicating less severe subendocardial myocardial ischaemia [27]. Total or subtotal vasospasm of a major coronary artery may result in ST-segment elevation in the leads corresponding to the distribution of that coronary artery. Other ECG changes associated with VSA include a delay in the peak of the R wave, an increase in the height and width of the R wave, a decrease in magnitude of the S wave, peak T wave, and/or negative U wave [11].

In order to identify or exclude potential aetiologies of MINOCA in patients suspected of VSA, the use of additional diagnostic tests is recommended. Echocardiography should be performed in the acute setting to assess regional wall motion abnormality (RWMA) or pericardial effusion. Computed tomography CAG may be considered for detection of atherosclerosis but does not identify plaque rupture or erosion. Cardiac magnetic resonance imaging allows the identification of RWMA, the presence of myocardial oedema or fibrosis/scar. An area of late gadolinium enhancement in the subendocardium suggests an ischaemic cause of injury (i. e. plaque disruption, vasospasm, thromboembolism or dissection), while a subepicardial localisation speaks in favour of cardiomyopathy (i. e. myocarditis or an infiltrative disorder) [28].

Non-invasive, non-pharmacological vasospasm testing showed a lower sensitivity compared with pharmacological testing [13]. Non-invasive IV ER testing using continuous monitoring of ST-segment deviation by ECG or RWMA by echocardiography to detect vasospasm-induced ischaemia in patients with near-normal angiographic findings has been described, but published data is limited. Ultimately, the cornerstone for the VSA diagnosis is based on provocative vasospasm testing with intracoronary administration of ACH or ER.

### Invasive evaluation

CAG during an episode of VSA most frequently shows vasospasm at a localised segment of an epicardial artery. However, multifocal, multi-vessel or diffuse vasospasm in one or multiple coronary arteries may occur. In most patients the location of vasospasm is fixed over time, but fluctuations from one vessel to another have been reported [29]. The frequency of multiple vasospasms during provocative vasospasm testing in Caucasians is 7.5%, markedly lower than in Japanese (24%) and Taiwanese populations (19%) [13, 30]. The angiographic criterion for ‘non-obstructive’ CAD (i. e. <50 stenosis) is hampered by the dynamic pathophysiological nature of an AMI, which may result in significant changes arising from fluctuating coronary vasomotor tone and the unstable coronary plaque [31]. In contrast, the finding of angiographically smooth coronary arteries does not preclude an aetiologic role of thrombotic disease, as intravascular ultrasound studies (IVUS) have demonstrated significant atherosclerotic burden in these patients [32]. IVUS or optimal coherence tomography may identify atherosclerotic plaque disruption, plaque erosion, coronary dissection or thrombosis.

### Indication and pharmacological agents for invasive provocative coronary vasospasm testing

Provocative coronary vasospasm testing has been used clinically for >40 years and should be undertaken in patients with suspected VSA if the diagnosis is to be pursued. It should be restricted to specialised centres and has been safely performed in patients with a recent AMI but must not be performed in the acute phase [33]. Tab. 3 summarises recommended indications for provocative testing as proposed by the COVADIS group [4]. We advise that provocative testing be performed in patients presenting with MINOCA unless there is a clear epicardial (plaque rupture, dissection), myocardial or non-cardiac cause. Although multiple provocative testing protocols have been developed to evaluate VSA, the gold standard method for provocative coronary vasospasm testing involves the administration of a provocative drug (typically ACH or ER) during CAG while monitoring patient symptoms, ECG and documentation of the coronary artery [4].

**Table 3:** Indications for provocative coronary artery spasm testing

*Class I (strong indication)*
History suspicious of vasospastic angina without documented episodes: – Nitrate-responsive rest angina – Marked diurnal variation in symptom onset/exercise tolerance – Rest angina without obstructive coronary artery disease – Unresponsive to empiric therapy
Presentation with acute coronary syndrome in the absence of a culprit lesion on angiography
Unexplained resuscitated cardiac arrest
Unexplained syncope with antecedent chest pain
Recurrent rest angina following angiographically successful PCI
*Class IIa (good indication)*
Invasive testing for non-invasively diagnosed patients unresponsive to drug therapy
Documented spontaneous episode of vasospastic angina to determine the ‘site and mode’ of spasm
*Class IIb (controversial indication)*
Invasive testing for non-invasively diagnosed patients responsive to drug therapy
*Class III (contra-indication)*
Emergent acute coronary syndrome
Severe fixed multi-vessel coronary artery disease including left main stenosis
Severe myocardial dysfunction
No symptoms suggestive of vasospastic angina

*PCI* percutaneous coronary intervention

The pharmacological agents most often used in provocative coronary vasospasm testing for the diagnosis of VSA are ACH and ER. Adverse reactions to ACH include hypotension, bradycardia, ventricular tachycardia, dyspnoea, and flushing [34]. Adverse reactions to ER are diverse and include angina, ischaemia/AMI, arrhythmia, nausea, allergic reaction, and ergotism [35]. The risks of invasive provocative vasospasm testing are low, as it allows rapid detection and treatment of the induced vasospasm. No deaths have been reported, although there is a 6.8% incidence of cardiac arrhythmias (i. e. comparable with that observed during spontaneous vasospasm episodes) [35]. Despite its high sensitivity, false negatives have been reported; therefore, a negative test cannot always exclude vasospasm [36].

### Invasive provocative coronary vasospasm testing protocol

At the Academic Medical Centre, University of Amsterdam, we perform provocative coronary vasospasm testing on a regular basis using intracoronary continuous infusion of ACH. The preparation of medication prior to provocative coronary vasospasm testing is summarised in Tab. 4. According to our institutional protocol, a 6 French sheath is inserted via the femoral or radial artery, whereafter patients are administered 70–100 IU/kg heparin. When using the radial approach the administration of vasospastic agents (‘radial cocktail’) is prohibited. During the entire procedure, the ECG and aortic pressure are monitored. After CAG has been performed, a Doppler guide wire (either FloWire or Combowire, both Volcano, Rancho Cardova, CA, USA) is introduced into the right or left coronary artery depending on the clinical presentation. The continuous flow measurement enables documentation of flow alterations such as early detection of reduction/cessation of blood flow due to EV and/or CMD. ACH is infused in continuous incremental doses until vasospasm is provoked (Tab. 4). If there are no signs of vasospasm, the coronary artery is visualised after each dose. If, before the third minute chest pain, ECG changes, arrhythmia or flow alterations detected by Doppler occur, the coronary artery is immediately visualised. If the criteria of vasospasm are fulfilled, the infusion of ACH is stopped and an immediate dose of nitroglycerin (200 µg) is administered intracoronarily. Visualisation of the coronary artery is repeated every minute to monitor the disappearance of the vasospasm. Finally, coronary flow reserve (CFR) measurement is performed (normal value CFR ≥2.5).

**Table 4:** Advice and dosing of medication for provocative coronary artery spasm testing

*Prior to procedure:*		
Withhold for 48 h	Long-acting calcium antagonists	
Withhold for 24 h	Caffeine	
	Long-acting nitrates	
	Short-acting calcium antagonists	
	α-Blockers	
	β-Blockers	
	ACE inhibitor	
	Angiotensin receptor blockers	
	Renin inhibitors	
	Aldosterone inhibitors	
Withhold for 4 h	Sublingual nitrates	
*During procedure:*		
Agent	Administration	*Dose*
– Acetylcholine	Intracoronary (manual) bolus injection	LCA: 20/50/100/200 µg RCA: 20/50/80 µg over 20 s with an at least 3‑min interval between each injection
	Intracoronary (continuous) infusion	LCA or RCA: Incremental doses of 0.288/2.88/28.8/288 µg during 3 min (maximal highest dose 864 µg)
– Ergonovine	Intracoronary (continuous) infusion	LCA: 16 µg/min during 4 min (maximal dose 64 µg) RCA: 10 µg/min during 4 min (maximal dose 40 µg)
	Intravenous (continuous) infusion	Incremental doses of 50/100/150 µg during 5 min

*ACE* angiotensin-converting-enzyme, *LCA* left coronary artery, *RCA* right coronary artery

### Diagnostic criteria for positive coronary vasospasm testing

**Fig. 2:**
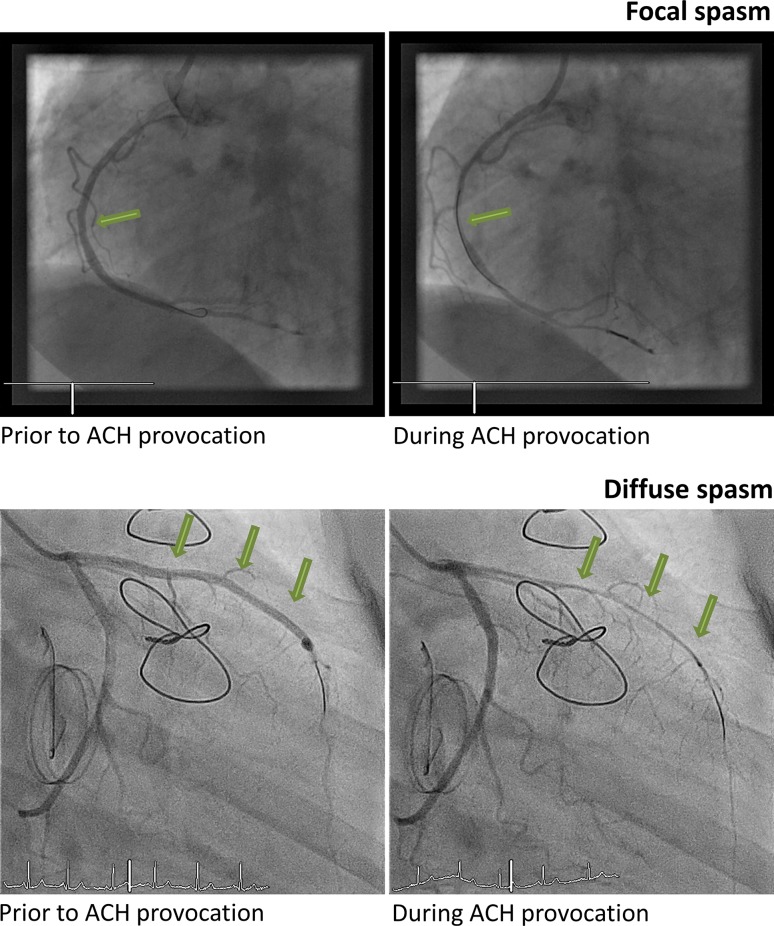
Epicardial coronary spasm. Example of focal coronary spasm (*upper panel*) and diffuse coronary spasm (*lower panel*) during provocative coronary vasospasm testing with intracoronary acetylcholine (*ACH*)

A positive response to ACH testing for EV is defined as the test inducing all of the following: (1) reproduction of the previously reported chest pain, (2) ischaemic ECG changes (ST-segment elevation or depression), and (3) >90% vasoconstriction on angiography (see Fig. 2).

**Fig. 3:**
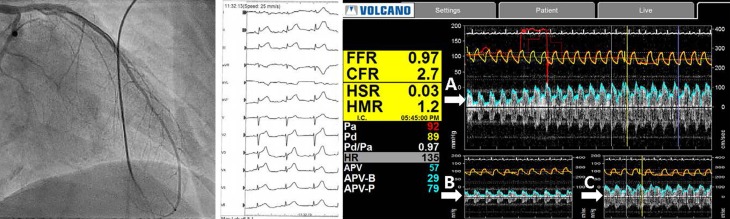
Coronary microvessel spasm. Example of coronary microvascular dysfunction. During infusion of intracoronary acetylcholine (IC ACH) there is no visible spasm of the left coronary artery (*left panel*). During infusion the ECG shows diffuse ST-segment elevation (*mid panel*). The coronary flow reserve (*CFR*) prior to IC ACH administration is 2.7 (*right panel*, *A*), a reduced CFR during IC ACH infusion (*right panel*, *B*), and recovery of the CFR after administration of nitroglycerin IC (*right panel,* *C*) (*FFR* fractional flow reserve, *HSR* hyperaemic stenosis resistence, *HMR* hyperaemic microvascular resistance)

A positive response to ACH testing for CMD due to microvascular spasm is defined as the test inducing the following: (1) reproduction of the previously reported chest pain, (2) ischaemic ECG changes, (3) no signs of EV (≥90% diameter reduction) but an evident reduction (i. e. cessation or slow) of coronary flow as measured with the Doppler flow wire, (4) normal microvascular function documented with a CFR of ≥2.5 and/or normal myocardial blush (see Fig. 3; [37]).

A negative response to ACH may provide evidence for CMD due to causes other than microvascular spasm, i. e.: (1) endothelial dysfunction (>20%, but <90% reduction in coronary luminal diameter during ACH provocation), (2) impaired epicardial and microvascular dilatation (CFR <2.5), (3) increased microvascular resistance (HMR ≥25, IMR >2.4), aside from EV and microvascular vasospasm [38].

## Treatment of patients with vasospastic angina

### Lifestyle adaptations

Before initiating pharmacotherapy, risk factor modification should be taken into consideration. Importantly, smoking cessation is one of the most compelling risk factors that can be modified [21]. Obese patients must be advised to lose weight. Exercise training, cardiac rehabilitation, and cognitive behavioural therapy are other important interventions. Excessive fatigue and mental stress must be avoided and patients should be advised to limit alcohol consumption. In addition, pharmacotherapy must aim at controlling blood pressure, impaired glucose tolerance and lipid abnormalities [39].

### Pharmacological treatment for VSA

Pharmacological treatment of VSA includes calcium channel blockers (CCBs) and non-specific vasodilators such as nitrates. The two different subclasses of CCBs, the dihydropyridines (DHP) and the non-DHP (diltiazem and verapamil), both induce vasodilation of the peripheral arteries and on the myocardium via inhibition of calcium influx through the L‑type calcium channels in excitable membranes [40]. As a result of extensive first pass metabolism, higher doses of non-DHP CCBs are recommended during initiation of therapy, an effect not observed with DHPs. Compared to short-acting CCBs, verapamil’s extended release has been shown to significantly improve symptoms of limited exercise tolerance, angina episode duration, and heart rate [40]. Adverse events associated with DHP are mainly caused by peripheral vasodilating properties, whereas negative chronotropic effects of non-DHP cause bradycardia and atrioventricular conduction delay, including second- and third-degree block [40]. Short-acting DHP can worsen cardiac outcomes through induction of reflex sympathetic activation leading to an increase in cardiac oxygen demand, tachycardia, and increased myocardial ischaemia [41]. Non-DHP can be harmful in patients with heart failure with reduced ejection fraction due to worsening of heart failure from their negative inotropic effects and should be prescribed with caution in elderly patients and those with chronic kidney disease due to suppression of the sinoatrial activity [40].

Nitrates dilate the coronary vasculature and reduce ventricular filling pressures through venodilation enhancing subendocardial perfusion of ischaemic areas in the myocardium [42]. However, the antianginal effect results mostly from the ability to decrease myocardial oxygen demand through systemic venodilation. Short-acting, sublingual nitroglycerin is preferred for acute angina, while long-acting nitrates are important for the chronic treatment of VSA, as they suppress acute angina attacks and may prevent recurrent attacks [43]. In practice, CCBs are preferred over long-acting nitrates, due to potential nitrate tolerance. However, combination therapy of a CCB and a nitrate may have a synergistic effect and provide relief when a patient has VSA refractory to monotherapy. Common adverse effects associated with the use of nitrates include headache, flushing, and tachycardia (which increases myocardial oxygen demand).

Low-dose aspirin (<100 mg daily) appears to be safe and may be effective in preventing acute attacks; nevertheless, robust data are lacking [44]. The use of high-dose aspirin (>325 mg daily) should be avoided as it may provoke exacerbations. Similarly, beta-blockers should be avoided as they can exacerbate VSA [45]. If a beta-blocker is absolutely indicated, labetalol or carvedilol may be considered because these agents possess mixed (alpha_1_- and beta-adrenergic receptor antagonist) properties which may result in overall vasodilation. It should also be noted that beta-blockers are indicated in cases of CMD, due to endothelial dysfunction and impaired vasodilation, in the absence of evidence of VSA [38]. Statins should be considered as the pleiotropic effects, such as antioxidant activity, may help attenuate vasoconstriction and prevent VSA [46]. Moreover, statins may help to improve endothelial function and thereby mitigate vasoconstriction. Currently, the effects of alpha-adrenergic receptors on coronary vasospasm have yet to be elucidated. Despite pharmacotherapy, refractory angina remains in 10–20% of patients.

### Invasive/non-pharmacological treatment

Invasive, non-pharmaceutical treatment includes stent implantation. However, it has been reported that vasoconstriction in segments adjacent to stents occurs in response to intracoronary ACH infusion. Partial sympathetic denervation can be considered in selected cases [8]. Thus far, there has been no sufficient evidence regarding the indication of implantable cardioverter defibrillators (ICDs) in survivors of OHCA with non-obstructive CAD in whom coronary vasospasm was induced during a provocation test. These patients should be treated with adequate pharmacotherapy, and physicians may consider ICD implantation for secondary prevention of cardiac arrest [47]. A recent study evaluated the long-term prognosis in Caucasian patients presenting with OHCA caused by coronary vasospasm [48]. All patients received a CCB or nitrates or both. With a mean follow-up of 7.5 ± 3.3 years, 2 out of 8 patients had an appropriate shock therapy.

## Conclusions

VSA is more common in female patients and remains a challenging diagnosis. In females, VSA may present with different symptoms than those in males. Particularly in patients with a MINOCA and/or suspected VSA, invasive provocative coronary vasospasm testing is recommended to confirm the diagnosis. Untreated VSA patients are at risk for major adverse cardiac events such as AMI, arrhythmias or SCD. Treatment should focus on lifestyle adaptation and pharmacotherapy with CCB with the addition of nitrates when symptoms remain.
